# Reduced shipping during COVID-19 enhanced the diurnal feeding activities of a small odontocete: implications of modern anthropogenic activities

**DOI:** 10.1093/nsr/nwae476

**Published:** 2024-12-27

**Authors:** Jiansong Qiu, Ding Wang, Songhai Li, Kexiong Wang, Zhigang Mei

**Affiliations:** The Key Laboratory of Aquatic Biodiversity and Conservation of the Chinese Academy of Sciences, Institute of Hydrobiology, Chinese Academy of Sciences, China; University of Chinese Academy of Sciences, China; The Key Laboratory of Aquatic Biodiversity and Conservation of the Chinese Academy of Sciences, Institute of Hydrobiology, Chinese Academy of Sciences, China; The Key Laboratory of Aquatic Biodiversity and Conservation of the Chinese Academy of Sciences, Institute of Hydrobiology, Chinese Academy of Sciences, China; Marine Mammal and Marine Bioacoustics Laboratory, Institute of Deep-sea Science and Engineering, Chinese Academy of Sciences, China; The Key Laboratory of Aquatic Biodiversity and Conservation of the Chinese Academy of Sciences, Institute of Hydrobiology, Chinese Academy of Sciences, China; The Key Laboratory of Aquatic Biodiversity and Conservation of the Chinese Academy of Sciences, Institute of Hydrobiology, Chinese Academy of Sciences, China

**Keywords:** small odontocete, diel feeding rhythm, shipping, reversible, Yangtze finless porpoise

Anthropogenic disturbances have led to an increase in nocturnal activities among terrestrial species [1], but their influence on aquatic mammals is still unknown. Among these, small odontocetes in freshwater and nearshore environments are facing significant human-induced pressures [2,3], particularly from prevalent and growing shipping traffic [4]. The ‘anthropause’ due to the COVID-19 lockdown provided a valuable opportunity to study the effect of shipping on the diel rhythms of small odontocetes. Our study found that the Yangtze finless porpoise (YFP; *Neophocaena asiaeorientalis asiaeorientalis*), a critically endangered freshwater odontocete, exhibited more diurnal feeding when shipping activity decreased during the lockdown. This finding was further supported by our controlled experiments. Although it is well understood that shipping is a critical factor in the endangerment of cetaceans, changes in diel rhythm remain an ongoing and ignored concern.

We reviewed the diel rhythms of small odontocetes and found that most species are likely to exhibit nocturnal activities (Fig. 1a, Supplementary Table S1). Specifically, among the 24 small odontocete species in our analysis of diel rhythms, 16 species were documented as exclusively nocturnal, seven species were recorded as both nocturnal and diurnal, while only one species was recorded as strictly diurnal. Notably, the habitat of the investigated odontocetes showed

**Figure 1.**
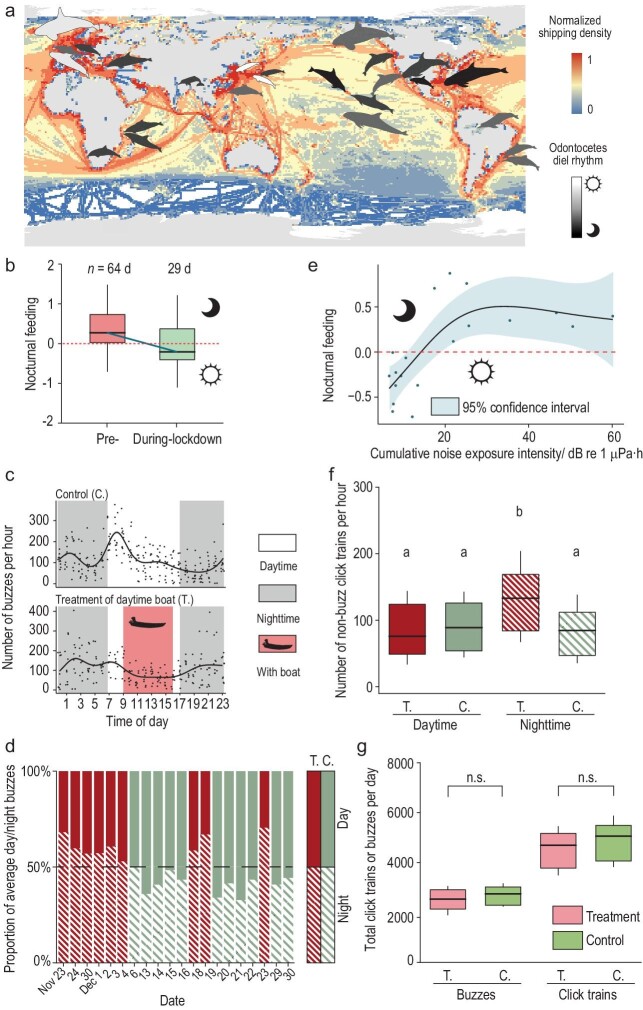
Diel rhythm of global small odontocetes and the effects of shipping on the Yangtze finless porpoise (YFP). (a) Diel rhythm of small odontocetes and global shipping density. Dolphins’ beaks point to the study areas. White represents diurnality and black represents nocturnality. (b) Diel feeding rhythm of wild YFPs reversed (*P* = 0.018) after shipping traffic was confined before and during the initial stage of COVID-19. The blue line connects the medians for YFPs’ diel feeding rhythm (>0 for nocturnal feeding, <0 for diurnal feeding) before and after shipping was limited. Boxes represent the first to third quartile of the data range, and the whiskers are the standard deviation. (c) In the control, the overall feeding frequency of YFPs was significantly higher during the day than at night (*P* < 0.001). By contrast, in the treatment of the daytime boat, the overall feeding frequency of YFPs was significantly lower during the day than at night (*P* < 0.001). (d) This trend remained consistent throughout three cycles of controls and treatments. (e) An increase in daytime cumulative noise exposure intensity (NEI_cum_) led to the manifestation of nocturnal feeding behavior in YFPs (*P* = 0.002, with *R_adj_*^2^ = 0.556, *n* = 19 days). (f) Nighttime non-buzz click trains were augmented in the presence of daytime shipping (*P* < 0.001). (g) Total click trains and buzzes per day remain similar in the presence and absence of daytime shipping. Review drawing number: GS 京 (2025) 0087号.

intensive shipping activities, and the effect of shipping on the diel rhythm of these species was generally overlooked. Therefore, we hypothesize that shipping may have a potential impact on the diel rhythms of small odontocetes. While there are numerous studies on how shipping affects their behavior [3,5,6], little attention has been given to how long-term shipping impacts the diel rhythms, particularly the feeding rhythms of small odontocetes.

To determine the extent of this impact, we conducted long-term monitoring of wild YFPs before and during the initial stage of the COVID-19 lockdown when shipping dramatically declined [7]. Previous studies showed that wild YFPs preferred nocturnal feeding [6]. YFPs use echolocation (click trains) to navigate and forage, similar to other odontocetes [8]. The buzz emitted by the YFPs, characterized by click trains with inter click intervals less than 10 ms, is considered as the indicator of their feeding behavior [6]. In this study, we quantified the YFP's feeding frequency based on the number of buzzes produced (see the supplementary materials for further methodological details). The monitoring results from three sites over a total of 93 days revealed a significant impact of shipping on the diel feeding rhythms of YFPs, with increased diurnal feeding during the ‘anthropause’ (Fig. 1b, Fig. S2). Furthermore, the number of vessels during the day and night was a significant predictor

of their diel feeding rhythms, suggesting that daytime-dominant shipping may alter the feeding patterns of YFPs. Thereby we hypothesized that: (i) YFPs may engage more in diurnal feeding in environments with reduced shipping, and (ii) YFPs in the wild engaged in more nocturnal foraging, possibly due to the pressure of diurnal shipping activities.

To verify the hypotheses, we further conducted controlled experiments in the conservation area (Fig. S1) with the absence or presence of daytime shipping as an explanatory variable. We set two hydrophones that could completely cover the swimming range of YFPs, therefore avoiding bias of spatial preferences. A total of 51 970 buzzes and 99 631 click trains from two YFPs were detected from 23 November to 30 December 2021. In the absence of shipping (control), the feeding frequency of YFPs was predicted to be 1.42 times (95% CI: 1.19–1.69) higher during the day than that at night [residual deviance: 287.88 on 262 df, *P* < 0.001, Akaike information criterion (AIC) 2966.6, generalized linear model (GLM)] (Fig. 1c). Two complementary monitorings in the absence of shipping in different areas (Fig. S1) and seasons proved the robustness of the results (see Fig. S3). In the presence of daytime shipping (treatment), on the contrary, the feeding frequency of YFPs was predicted to be 0.65 times (95% CI: 0.54–0.79) lower in the day than at night (residual deviance: 225.93 on 207 df, *P* < 0.001, AIC 2316.4, GLM) (Fig. 1c). This means that YFPs increased the proportion of nocturnal feeding by 1.47 times in response to daytime shipping. However, after the removal of shipping disturbance, the diel feeding rhythm of YFPs quickly returned to its pre-shipping state (Fig. 1d), and the overall difference in the day/night buzz ratio remained significant (Table 1). Furthermore, a significant correlation between daytime shipping-induced noise (Fig. S4) and YFPs' diel feeding rhythm was demonstrated by the generalized additive models (GAMs) with Gaussian family (Fig. 1e). An increase in the cumulative noise exposure intensity (NEI_cum_) caused by diurnal shipping noise led to a reversal of the YFPs' diel feeding rhythm, making them become more nocturnal (*P* = 0.002, edf = 2.406. GAM explained 61.6% of deviance, with *R_adj_*^2^ = 0.556, *n* = 19 days).

**Table 1.** GLM analyzing diel feeding rhythm of YFPs among different group sets in controlled experiments. Values of nocturnal feeding were calculated by the logarithm of buzz ratio night : day. Nocturnal feeding >0 represents nocturnal feeding trends, while <0 represents diurnal feeding trends.

Dates	Presence of boat	Nocturnal feeding	Number of days	*P*	AIC
23–24 Nov, 30 Nov–3 Dec	Present	0.359	6	0.001	1546.3
4 Dec, 6 Dec, 13–15 Dec	Absent	−0.266	5	0.036	1374.3
16 Dec, 18 Dec	Present	0.409	2	0.084	500.7
19–22 Dec	Absent	−0.496	4	<0.001	1071.7
23 Dec	Present	0.873	1	0.005	274.1
29–30 Dec	Absent	−0.299	2	0.177	519.1
Overall	Present	0.426	9	<0.001	2316.4
Overall	Absent	−0.352	11	<0.001	2966.6

Our findings of the increased diurnal feeding in environments with reduced shipping suggest that this feeding pattern can bring a number of advantages for YFPs. Firstly, although odontocetes were believed to rely on echolocation for foraging, vision may still play a crucial role when approaching prey [8,9], making daytime hunting more efficient. Secondly, because the primary preys of YFPs consist of small and diurnal fish species in the upper and middle water layers [10], synchronizing with the activity patterns of the prey is also essential for efficient hunting. Finally, YFPs may mainly rest at night, similar to other odontocete species [11].

Nevertheless, the increasing of diurnal shipping and noise could alter this rhythm, causing YFPs to adopt a nocturnal foraging strategy, which may be explained by the following reasons. Firstly, shipping noise may directly distract YFPs or serve as a source of fear, thus affecting the efficiency of daytime hunting [12]. Secondly, shipping activities may disturb fish schools [13], thus indirectly affecting YFPs’ feeding rhythms by altering prey distribution.

However, the increase in nocturnal foraging of YFPs will lead to additional energy consumption and increase survival costs. In the controlled experiments, the total feeding attempts in a day stayed constant (Fig. 1g), indicating that energy intake may not be limited. However, the non-buzz echolocations increased with the emergence of nocturnal feeding rhythms (Fig. 1f), suggesting that YFPs may spend more effort on echolocating to ensure successful prey capture during the night. This could be explained by acoustic compensation for visual loss at night, and thereby would bring more energy consumption than diurnal feeding. In addition, although disturbances were reduced at night, changes in the density and species of nocturnal prey may have increased the difficulties of predation.

We also found that the diel feeding rhythm of wild YFPs could still be restored after the shipping traffic was confined, even once they had coexisted with shipping noise for a long time. This reveals a high plasticity on their rhythm formation. Importantly, the plasticity might be determined jointly by human activities and the natural environment. We believe that the diurnality exhibited by YFPs in the absence of disturbance could be an intrinsic rhythm. However, on the other hand, this plasticity on diel feeding rhythm raises the possibility that the rhythmic change may not involve the circadian system [14], which means the animals have undergone long-term pressure rather than adapted to the temporal distribution of shipping traffic. It is common for species to expand their territories and restore behavior among environments with reduced anthropogenic disturbance [15]. This variability brings hope for YFPs undergoing rhythmic changes and highlights an enlightenment for the conservation of cetaceans. For example, vessel speed limitations and shipping regulations that effectively reduce underwater noise should be fully applied in cetaceans’ key habitats and feeding grounds [5].

To the best of our knowledge, we are reporting the first case of human activity altering the diel feeding rhythm of cetacean species. Although it is well understood that shipping can cause cetaceans’ avoidance reactions in space, we show here that the uneven distribution of shipping in time can elicit change in diel rhythm. Over the past two centuries since the Industrial Revolution, shipping and underwater noise have gradually altered the underwater soundscape [3]. Avoiding busy daytime shipping traffic, as an adaptive survival strategy for YFPs, may reduce collision risks and partially compensate for missed prey during the day. Nocturnality, however, necessitates more energy on foraging and fitting in changes of prey species and density. This suggests that changes in diel feeding rhythm may pose potential survival threats to these aquatic mammals as well. Because their rhythmic change can be ‘easily’ restored through measures such as vessel speed limitations and shipping regulations, we therefore urge policies on shipping management being implemented to help mitigate the rhythm changes and enhance survival capabilities. In addition, we call for global and precise studies of the relationships between shipping and the diel rhythm of cetaceans, especially for species in polar regions under rapid global changes.

## Supplementary Material

nwae476_Supplemental_File
