# Supplementary material for: Reduced shipping during COVID-19 enhanced the diurnal feeding activities of a small odontocete: implications of modern anthropogenic activities
Source: Natl Sci Rev. 2024 Dec 27;12(3):nwae476. doi: 10.1093/nsr/nwae476 (PMC11846081; doi:10.1093/nsr/nwae476)
Supplement: nwae476_Supplemental_File [file nwae476_supplemental_file.docx]

**Supplementary Information for**

Reduced shipping during COVID-19 enhanced the diurnal feeding activities of a small odontocete: implications of modern anthropogenic activities

Jiansong Qiu, Ding Wang, Songhai Li, Kexiong Wang, and Zhigang Mei

Corresponding Author: Songhai Li, Kexiong Wang and Zhigang Mei.

Email: lish@idsse.ac.cn (S.L.); wangk@ihb.ac.cn (K.W.); meizhigang@ihb.ac.cn (Z.M.)

**This PDF file includes:**

Materials and Methods

Figures S1 to S5

Tables S1

References for SI reference citations

**Materials and Methods**

**Literature review on the diel rhythm of small odontocetes**

To understand the potential relationships between diel rhythms of global small odontocetes and shipping, we collected data from published literature (Supplementary Fig. 1, Supplementary Table 1). We used the following search terms (matching topic): *(****species*** ***scientific name*** OR ***species*** ***Latin name****)* AND *(feeding* OR *forag** OR *activit** OR *prey** OR *diet* OR *food)* AND *("circadian rhythm"* OR *diel** OR *nocturnal** OR *diurnal** OR *day* OR *night)*. We determined small odontocetes as species included in families *Delphinidae*, *Phocoenidae*, *Platanistidae*, *Iniidae* and *Pontoporiidae*. We searched the published literature using the Web of Science databases. We included all studies that contained data on 24-h click train or buzz variations for specific species. If the original study divided day and night, we followed the authors’ classification for day and night. If day and night were not clarified, we then defined day as the time from sunrise to sunset, and night from sunset to sunrise. Sunrise and sunset times for the reported study areas were checked out from the website: https://www.timeanddate.com/. For each study, we defined each species’ diel rhythm (DR, Eq. 1) as follows:

$$\begin{aligned} DR=\ln\frac{{Vocalization}_{day}}{{Vocalization}_{night}}\#\left( 1 \right) \end{aligned}$$

where Vocalization_day_ is daytime buzz or click train per hour, and Vocalization_night_ is nighttime buzz or click train per hour. A positive DR value means nocturnality and negative NF value means diurnality.

Shipping density data in Fig. 1A (at 1-degree resolution, 2015) was adapted from Wu *et al* [1], and silhouettes are from Jefferson *et al* [2].

**Experimental data collection and data processing**

Controlled experiment in *Laowan* was a part of the plan of reintroduction of the Yangtze finless porpoise, which was approved and authorized by the Ministry of Agriculture and Rural Affairs of the People's Republic of China. All the acoustic monitoring in conservation areas or field were conducted without posing any behavioral impact on YFPs. Due to YFPs' underwater and wide-ranging nature, combined with the turbid waters of the Yangtze River, direct observation and long-term tracking are challenging. High frequency click trains emitted by YFPs are typically monitored using underwater recording devices to represent their occurrence, distribution, and behavior [3, 4]. Click train with ICIs less than 10 ms, also known as buzz, is believed as feeding attempts [5, 6].

***Field studies***

Acoustic data loggers C-POD and F-POD were utilized to record YFP echolocations. These instruments can record sounds within 20-160 kHz in the environment, which include the echolocation frequency range of YFP. C-POD/ F-POD were placed in each of the following locations: *Duchang* in Poyang Lake, *Hukou* in the confluence area of Poyang Lake and the Yangtze River, and *Pengze* in the mid-lower reaches of the Yangtze River (Fig. 1C and D). The devices were secured on steel frames underwater at a depth of 1.5 meters near maritime vessels or docks. The monitoring period for the *Duchang* was Jan 2, 2020 - Mar 14, 2020 (data deficiency during Feb 4 and Feb 11), for Hukou was Jan 14, 2020 - Mar 12, 2020, and for *Pengze* was Jan 18, 2020 - Mar 20, 2020. During the initial period of COVID-19 pandemic lockdown (Feb 1, 2020 - Feb 28, 2020), shipping traffic was limited near the monitoring sites. The number of vessels per hour passing within a 1000-meter radius around the monitoring sites was obtained from the China Maritime Safety Administration.

Data processing was performed using C-POD.exe v2.048 (Chelonia Limited, Mousehole, Cornwall, UK) and F-POD.exe v091.0.0.17 (Chelonia Limited, Mousehole, Cornwall, UK). Both of which are equipped with built-in programs, KERNO classifier, for automatic analysis and selection of YFP echolocations, specifically targeting buzzes. We calculated YFP’s nocturnal feeding tendency (NF; Eq. 2) as follows:

$$\begin{aligned} NF=\ln\frac{{Buzz}_{night}}{{Buzz}_{day}}\#\left( 2 \right) \end{aligned}$$

where Buzz_night_ is nighttime buzz per hour, and Buzz_day_ is daytime buzz per hour. A positive NF value means nocturnal feeding and negative NF value means diurnal feeding.

***Controlled experiments in protecitve area Laowan***

The controlled experiments in protective area *Laowan* employed the use of the Soundtrap 300HF hydrophone (Ocean Instruments Ltd, New Zealand) to record the sounds produced by YFPs and underwater noise. *Laowan* is a part of the *Xinluo Baiji National Nature Reserve* in the middle reaches of the Yangtze River within Hubei Province. *Laowan* water is a closed area of approximately 3 km in length and 70 m in width. In 2021, there were 2 male YFPs living in this area. These individuals were born in an *ex situ* reserve, *Tian'ezhou*, and were transported to *Laowan* water for pre-release acclimatization training. Previous research [7] indicates that these individuals have successfully adapted to *Laowan*, suggesting that their conditions during the experiment were similar to those of other individuals. From Nov 23 to Dec 30, 2021, we deployed two Soundtraps at positions 1 km upstream and downstream in the water (Fig. S1B), ensuring full coverage and reducing monitoring overlap. The Soundtrap parameters were set as follows: Sample Rate of 576 kHz, High gain, High Pass Filter Off, and continuously record. The devices were installed underwater at a depth of 2 m and 15 m from the shore using floats, anchors, and ropes. Due to rapid battery consumption during continuous recording, the devices must be retrieved weekly to export data, recharge, and reinstall.

The control group was conducted from Dec 4-15, Dec 19-22, and Dec 29-30, 2021, during which time the water area remained quiet with complete isolation from boats. The treatment group was conducted from Oct 23-Nov 3, Nov 16-18, and Nov 23, 2021. We introduced boat treatments to simulate the shipping traffic of the Yangtze River. The types of boats included small powerboat and large cargo vessel (Fig. S5), with operating time from 9: 00 to 16: 00. In each set of treatments: vessels continuously moved towards YFPs until YFPs crossed in the opposite direction and fled 500 m away from the vessel, then turned around and continued; The operations were repeated for 30 minutes, followed by 1-hour interval (engine off). During the daily treatments, 3-4 sets were repeated, and after conducting the experiments for 1-3 days, there was a break of several days. To quantify the effect of boat on YFPs’ diel feeding rhythm, a natural day was defined as Day 1 sunrise to Day 2 sunrise in this experiment, not 0:00 to 24:00. The missing days in both groups were excluded due to interference on shore on those specific days.

Soundtrap-recorded audio was obtained by downloading and decompressing sud. files via Soundtrap Host 4.0. A custom program [8] written in Matlab R2021b (Math Works, Natick, MA) was used to help recognize YFP click trains and buzzes. Additionally, the root mean square sound pressure level (SPLrms) per hour of underwater noise was calculated, while filtering below 80 kHz was applied to exclude YFP-generated sounds. Cumulative noise exposure intensity (NEI_cum_; Eq. 3, Fig. S4) was defined calculating the time integral of the part where SPL_rms_ exceeds the background SPL_rms_ of a day [9]:

$$\begin{aligned} {NEI}_{cum}=\sum_{i\in time of day} \left( {SPLrms}_{i}-{SPLrms}_{background} \right)\times tdB re 1\mu Pa\cdot h\#\left( 3 \right) \end{aligned}$$

where SPLrms_i_ is SPL_rms_ of the hour, SPLrms_background_ is the minimum SPLrms of the day, and *t* in the equation is defined as 1 hour. Buzzes per hour were divided into groups “day” and “night” based on sunrise and sunset time from the website: <https://www.timeanddate.com/>.

***Complementary monitoring in different seasons and areas***

We further conducted mornitoring in absence of boats in another protective area *Tian'ezhou* oxbow and in summer of *Laowan*. *Tian'ezhou* is located approximately 180 km upstream of *Laowan*. It is the largest ex situ reserve for YFPs in China, covering an area of about 13.7 km². It is also situated within Hubei Province. The oxbow is home to about 100 YFPs. According to the distribution patterns of YFPs in *Tian'ezhou* oxbow, we evenly deployed 5 Soundtraps in YFP high-density areas (Fig. 1A) from Dec 8 to Dec 25, 2022. Each device was placed 1 km apart to ensure full coverage and reduce monitoring overlap. Besides, 1 Soundtrap was placed at the center of the water in *Laowan* from May 27 to Jun 16, 2023. All the Soundtraps parameters were set as follows: Sampling rate of 576 kHz, High gain, High Pass Filter Off, recorded for a period of 30 minutes once every 60 minutes. Soundtraps were anchored underwater at a depth of 2 m and 15 m from the shore using floats, anchors, and ropes. They were retrieved after being placed for 2 weeks.

**Statistical analysis**

Data analyses were carried out using R: A language and Environment for Statistical Computing version 4.1.3 (R Core Team 2013).

To verify the changes in wild YFPs’ diel feeding rhythm when shipping reduced in field studies, we divided dates into high-disturbed and low-disturbed periods according to the variation of boat number. Changes of YFPs’ diel feeding rhythm between high-disturbed and low-disturbed periods were then compared through Mann-Whitney U Test. We included dates when buzz occurred every hour in a day to ensure credible diel rhythm, and site *Duchang* was excluded due to the incomplete data. To further test for effects of ships on YFPs’ diel feeding rhythm, we used linear mixed model fit by REML in the R package *lme4* and *lmerTest*, with daytime ships per hour and nighttime ships per hour as fixed effects, and monitoring site as a random effect. AIC was used to get the best fit.

To test for relationships between time and buzzes in controlled experiments, we used generalized additive models (GAMs) in the R package *mgvc* with Poisson distribution. Differences on number of buzzes between day and night were further compared based on generalized linear model (GLM) with negative binomial distribution. To model the impact of daytime boat noise on diel feeding rhythm of YFPs, we used NEI_cum_ (Eq. 3) of underwater noise to quantify the effect of boat noise exposure. After initial data exploration, YFP’s nocturnal feeding (Eq. 2) was modeled as a function of daytime NEI_cum_ using GAMs with gaussian. Kruskal-Wallis test was used afterwards to test for the variation in sound composition of YFPs.

**Supplementary Figures and Tables**


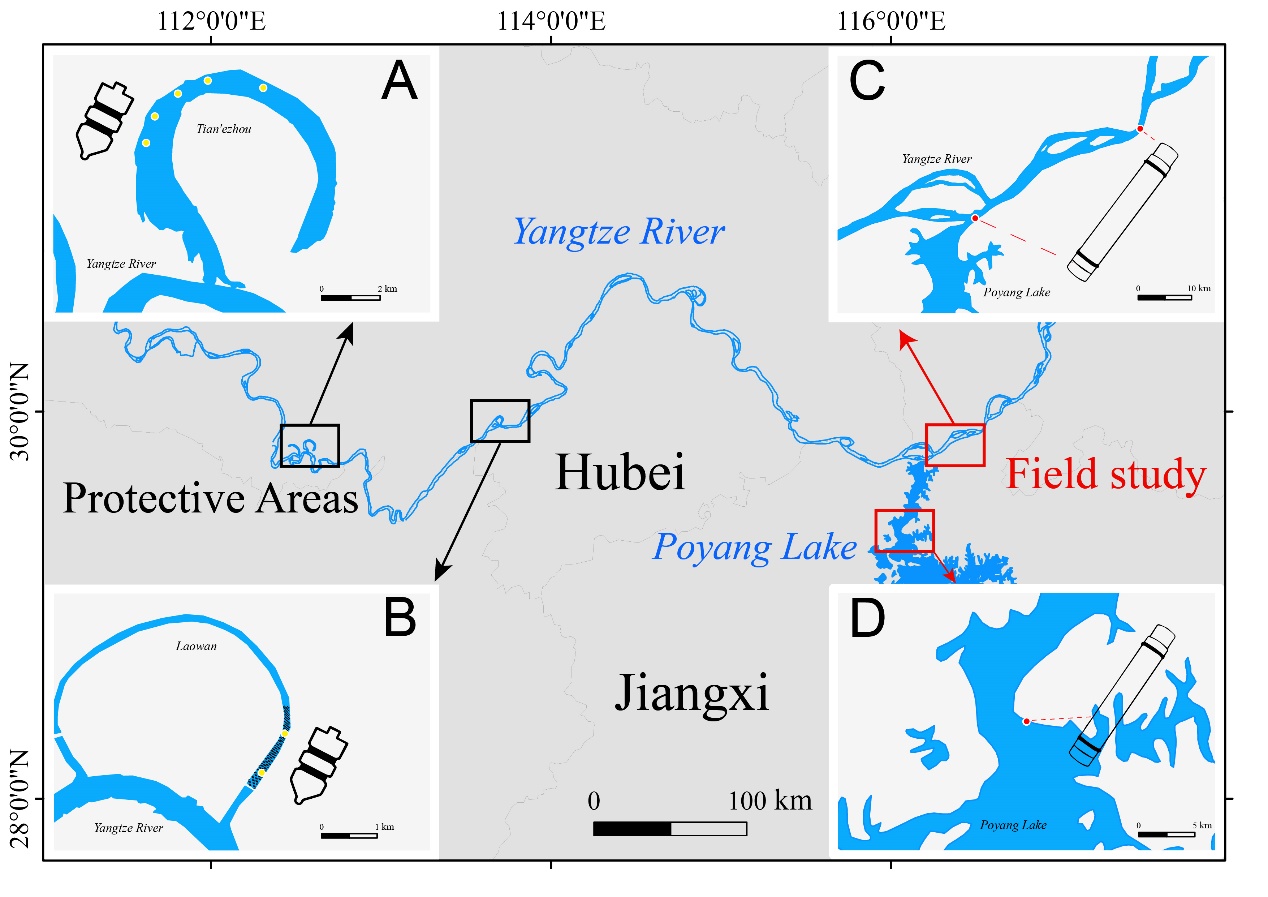


**Fig. S1. Study areas for field studies and controlled experiments.** (**A**) Protective area *Tian’ezhou* oxbow. (**B**) Protective area *Laowan*. The black shaded area represents the actual water body during the monitoring period. The deployment locations of Soundtraps are marked with yellow dots. (**C**) *Hukou* at the confluence of the Yangtze River and Poyang Lake, and *Pengze* in the mainstream of the Yangtze River. (**D**) *Duchang* in Poyang Lake. The deployment locations of C-POD/ F-POD are marked with red dots.

**
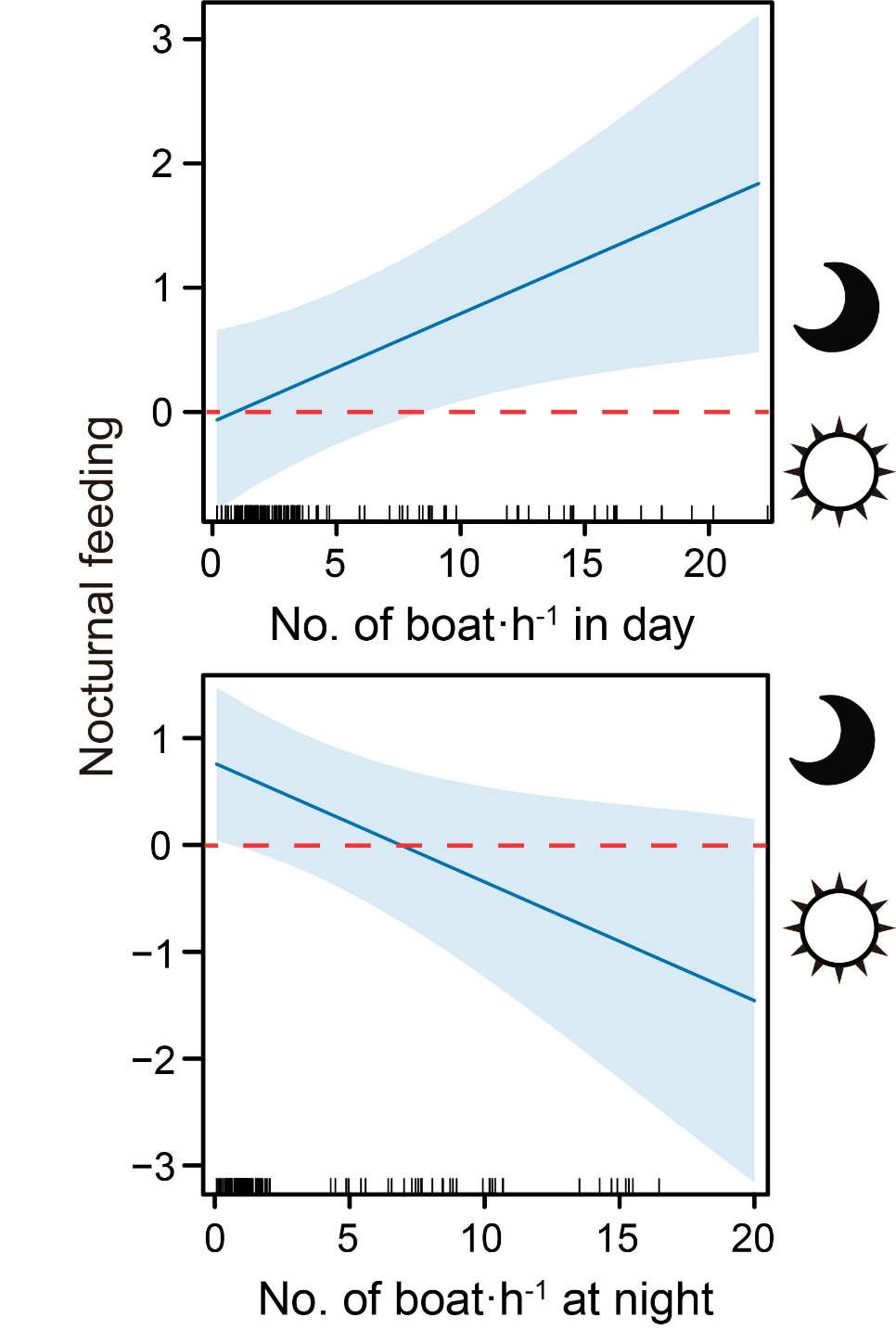
**

**Fig. S2. Effects of shipping on YFPs’ diel feeding rhythm in field studies** **before and during initial stage of COVID-19.** Wild YFPs tended to exhibit nocturnal feeding as daytime ship number increased. Nighttime ships were negatively correlated with the nocturnal feeding.


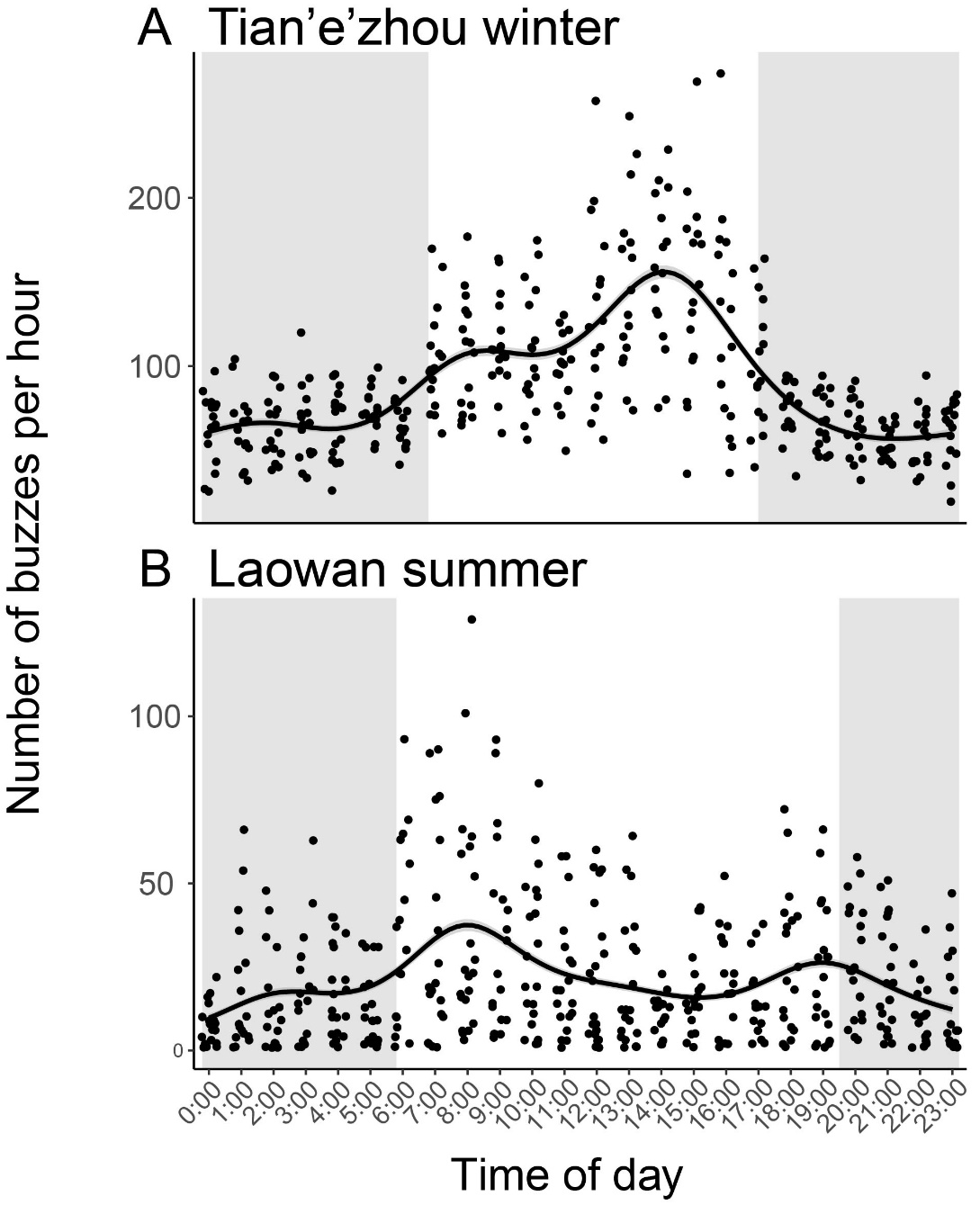


**Fig. S3. Diel feeding rhythm of YFPs in *Tian’ezhou* winter and *Laowan* summer in the absence of boat.** (**A**) *Tian’ezhou* winter: The feeding frequency of YFPs was 1.89 times (95% CI: 1.76-2.02) higher during the day than at night (df = 384, *P* < 0.001, AIC 3655.6, GLM). The interaction between time and buzzes was also significant: *P* < 0.001, edf = 8.884, GAM explained 52.1% of deviance, with *R_adj_*^2^ = 0.479. *n* = 384 hours. (**B**) *Laowan* summer: The feeding frequency of YFPs was 1.51 times (95% CI: 1.26-1.80) higher during the day than at night (df = 444, *P* < 0.001, AIC 3626.7, GLM). The interaction between time and buzzes was also significant: *P* < 0.001, edf = 6.074, GAM explained 10.5% of deviance, with *R_adj_*^2^ = 0.0926. *n* = 446 hours. Grey background represents nighttime and white background represents daytime


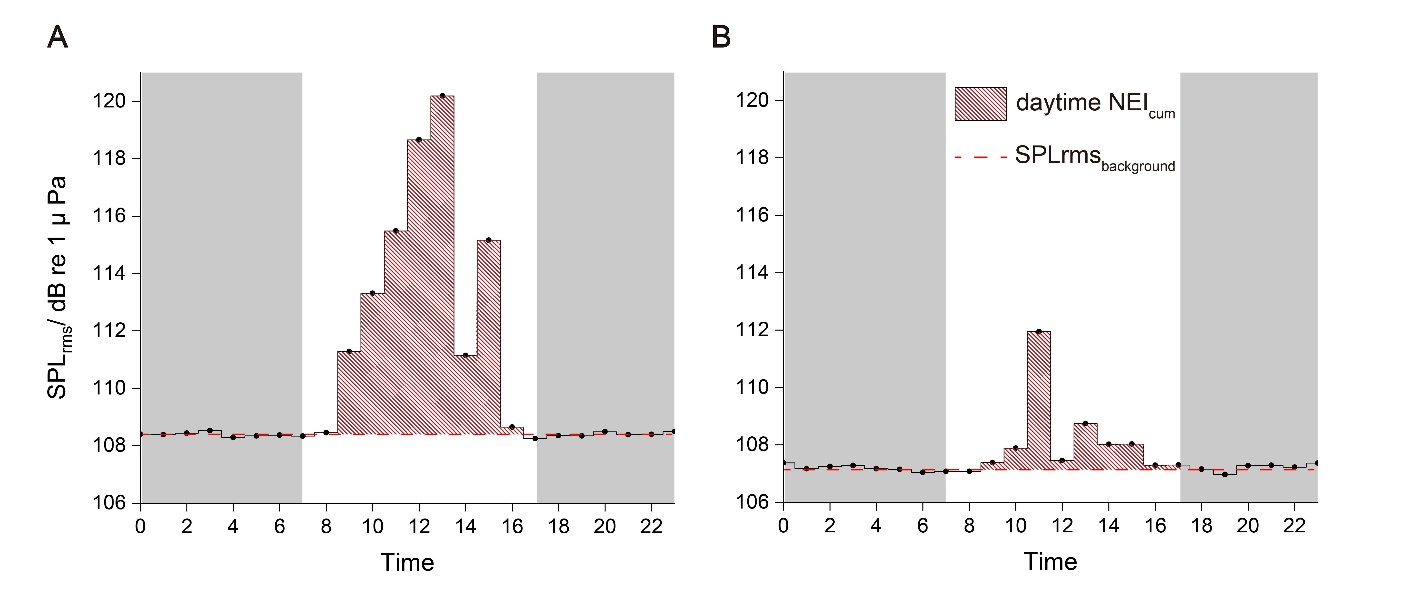


**Fig. S4. Examples of variation of day-night sound pressure level in treatment (A) and control (B).** Grey background represents nighttime, while white background represents daytime.


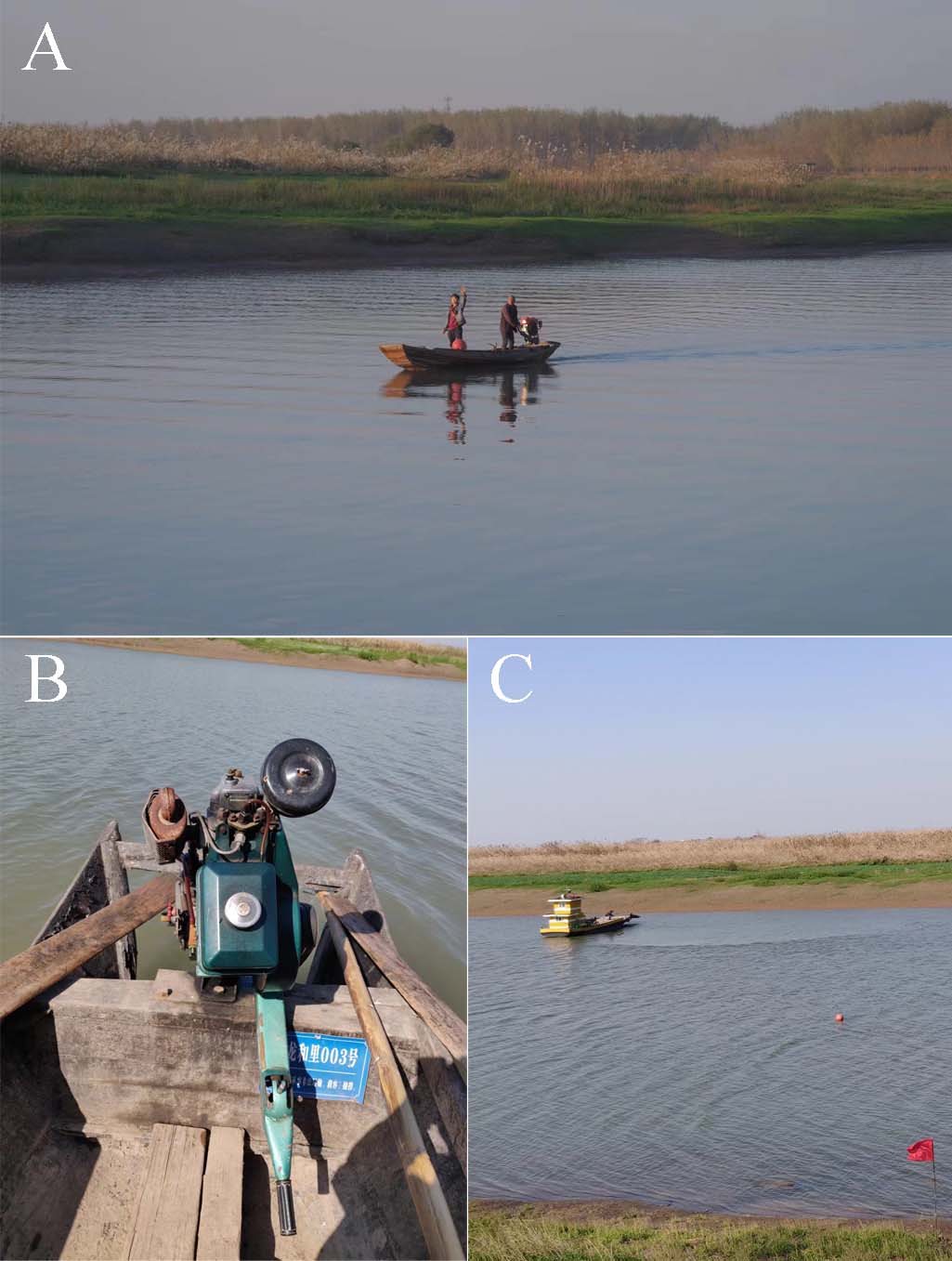


**Fig. S5. *Laowan* experiments with treatment of boat.** Two types of boats were used in the experiment, (**A**) powerboat and (**C**) cargo vessel (Soundtrap was attached under the red float on middle of water). (**B**) shows the engine of powerboat.

**Table S1. Diel rhythm of small** **odontocetes.** A negative Day/night ratio represents nocturnality, while a positive Day/night ratio represents diurnality. Day/night ratio was calculated by the logarithm of either buzz or click trains per hour during day/night. NA, not available.

| **Species** | **Diel rhythm** | **Day/night ratio** | **Methods** | **Longitude and Latitude** | **References** |
| --- | --- | --- | --- | --- | --- |
| Irrawaddy dolphin  *Orcaella brevirostris* | diurnal | NA | Binoculars | 13°56′ N, 105°56′ E | [10] |
| Killer whale  *Orcinus orca* | diurnal | 0.38 | Ecological  acoustic recorder (EAR) | 64°57′ N,  23°07′ W | [11] |
|  | nocturnal | -0.25 | Marine Autonomous Recording Unit (MARU) | 57° N,  170° W | [12] |
| Long-finned pilot whale  *Globicephala melas* | nocturnal | NA | A tag contains a VHF radio (Telonics) and a time-depth recorder (TDR-Mk6) | 43°17′ N,  7°30 E | [13] |
|  | diurnal | 0.19 | AMARs | 49° - 53° N,  10° - 16° W | [14] |
| Pygmy killer whale  *Feresa attenuata* | nocturnal | -3.22 | SPLASH10-268D Finmount tags | 27° - 29° N,  88° - 91° W | [15] |
| Melon-headed whale  *Peponocephala electra* | nocturnal | -0.26 | High-frequency acoustic recording package (HARP) | 5°52′ N, 162° 10′ W  5°52′ N, 162° 09′ W | [16] |
|  | nocturnal | NA | Depth transmitting satellite tags (Wildlife Computers SPLASH10-A in the LIM-PET configuration) | 20.06 N,  154.93 W | [17] |
| Guiana dolphin  *Sotalia guianensis* | nocturnal | -0.37 | Hydrophone C53 and digital recorder | 25°01′ S - 25°13′ S,  47°52′ W - 48°06′ W | [18] |
| Indian Ocean humpback dolphin  *Sousa plumbea* | nocturnal | -1.43 | C-PODs | 6.38° S,  39.37° E | [19] |
| Indo-Pacific humpback dolphin  *Sousa chinensis* | nocturnal | -0.33 | SoundTrap 300 HF | 22°16′36′′ N, 113°48′18′′ E | [20] |
|  | nocturnal | NA | SoundTrap 300 HF | 22°3′ N,  113°30′ E | [21] |
|  | nocturnal | -0.48 | Passive acoustic monitor (PAM) | 22.118° N, 113.716° E | [22] |
| Rough-toothed dolphin  *Steno bredanensis* | nocturnal | -2.41 | SPLASH10-A | 21.75° - 22.25° N,  159.75° - 160.25° W | [23] |
| Pacific white-sided dolphin  *Lagenorhynchus obliquidens* | nocturnal | -0.10 | HARPs | 32°30′N, 119°12′ W  34°15′N, 120°48′ W | [24] |
|  | nocturnal | -0.80 | Ocean Sonics icListen SC35 hydrophone | 48°25.6457′ N, 126°10.4799′ W | [25] |
| Risso’s dolphin  *Grampus griseus* | nocturnal | -0.97 | HARPs | 33°36′ N, 118°36′ W | [26] |
|  | nocturnal | NA | PAM | 37°02.087′ N, 13°51.733′ W | [27] |
|  | crepuscular | NA | EK 60 echosounders  split-beam Simrad EK60s at38 and 120 kHz (AUV) | 33.42 N,  118.21 W | [28] |
| Common bottlenose dolphin  *Tursiops truncatus* | nocturnal | -0.76 | A-tag | 41.1 N, 29.05 E | [29] |
|  | crepuscular | NA | T-PODs | 45°28′ S, 167°09′ E | [30] |
|  | diurnal | NA | Recording devices | 24.15 N,  110.34 W | [31] |
| Indo-Pacific bottlenose dolphin  *Tursiops aduncus* | nocturnal | -1.43 | C-POD | 6.38° S,  39.37° E | [19] |
| Pantropical spotted dolphin  *Stenella attenuata* | nocturnal | -0.43 | TDRs | 5° - 20° N,  85° - 105° W | [32] |
|  | nocturnal | 0.65 | A tag contains a VHF radio (MOD-125， Telonics) and a time-depth recorder (TDR-Mk6) | 20.7° - 20.8° N,  156.6° - 156.8° W | [33] |
| Spinner dolphin  *Stenella longirostris* | diurnal | 2.28 | EARs | 03°51′ S,  32°25′ W | [34] |
|  | nocturnal | -0.87 | EARs | 20.7 N, 156.7 W | [35] |
| Striped dolphin  *Stenella coeruleoalba* | nocturnal | NA | Stomach contents | 20° N, 110.2° W  10° N, 130.4° W  3° N, 95.5° W  4° S, 95.2° W | [36] |
| Common dolphin  *Delphinus delphis* | nocturnal | NA | Sony TCD-D10 ProII recorder | 52 N, 5.5 W | [37] |
| Heaviside’s dolphin  *Cephalorhynchus heavisidii* | nocturnal | -0.89 | T-POD | 22.87 N,  14.45 E | [38] |
| Harbor porpoise  *Phocoena* | nocturnal | NA | T-POD and C-POD | 54.5 N, 7.5 E | [39] |
|  | nocturnal | NA | C-POD | 51°35′ N,  3°55′ W | [40] |
|  | nocturnal | NA | C-POD | 55.46 N,  10.65 W | [41] |
|  | nocturnal | NA | T-POD | 54°30′ N,  11°20′ E | [42] |
|  | nocturnal | -0.09 | C-POD | 54.28 N, 9.89 W | [43] |
|  | nocturnal | -1.24 | T-POD | 55°41′ N,  4°05′ E | [44] |
|  | nocturnal | NA | A-tag W20-AS | 55° - 57° N,  10° - 13° E | [45] |
|  | nocturnal | NA | C-POD | 55°15′ - 55°25′ N,  10°50′ - 11°00′ E | [46] |
| Vaquita  *Phocoena sinus* | nocturnal | -1.56 | C-POD | 30.9° - 31.4° N,  114.4° - 114.8° W | [47] |
| East-Asian finless porpoise  *Neophocaena asiaeorientalis sunameri* | diurnal | 1.30 | SoundTrap 300HF | 37.3564° N,  119.4956° E | [48] |
|  | diurnal | 1.03 | A-tag,  SoundTrap 300HF | 33°51.151 N, 132°06.893 E | [49] |
|  | nocturnal | -0.78 |  | 34°41.833 N, 136°59.555 E |  |
| Ganges River dolphin  *Platanista gangetica* | nocturnal | -0.78 | A-tag | 28º16′16′′ N, 78º9′4′′ E | [50] |
| Amazon river dolphin  *Inia geoffrensis* | nocturnal | -0.46 | A-tag | 3°35′ S,  64°45′ W | [51] |
| Franciscana  *Pontoporia blainvillei* | nocturnal | -0.06 | C-POD | 27.25 S,  48.67 W | [52] |

**Supplementary References**

1. Wu L, Xu Y, Wang Q *et al.* Mapping Global Shipping Density from AIS Data. *Journal of Navigation*. 2016; **70**(1): 67-81. doi: 10.1017/s0373463316000345

2. Jefferson TA, Webber MA, Pitman RL *et al.* *Marine mammals of the world: a comprehensive guide to their identification. Second edition*2015.

3. Madsen PT, Siebert U, Elemans CPH. Toothed whales use distinct vocal registers for echolocation and communication. *Science*. 2023; **379**: 928–933. doi: 10.1126/science.adc9570

4. Akamatsu T, Wang D, Wang K *et al.* Biosonar behaviour of free-ranging porpoises. *Proc Biol Sci*. 2005; **272**(1565): 797-801. doi: 10.1098/rspb.2004.3024

5. Wang Z, Akamatsu T, Wang K *et al.* The Diel Rhythms of Biosonar Behavior in the Yangtze Finless Porpoise (Neophocaena asiaeorientalis asiaeorientalis) in the Port of the Yangtze River: The Correlation between Prey Availability and Boat Traffic. *PLoS ONE*. 2014; **9**(5): 1-12. doi: 10.1371/journal.pone.0097907

6. Vance H, Madsen PT, Aguilar de Soto N *et al.* Echolocating toothed whales use ultra-fast echo-kinetic responses to track evasive prey. *eLife*. 2021; **10**: e68825. doi: 10.7554/eLife.68825

7. Qiu J, Sun X, Wang D *et al.* The first case of reintroduction and behavioral adaptability of Yangtze finless porpoise. *Acta Hydrobiologica Sinica*. 2023; **47**(10): 1709-1718. doi: 10.7541/2023.2023.0064

8. Li W, Qiu J, Lei P *et al.* A real-time passive acoustic monitoring system to detect Yangtze finless porpoise clicks in Ganjiang River, China. *Frontiers in Marine Science*. 2022; **9**: 883774. doi: 10.3389/fmars.2022.883774

9. Outi M. Tervo SBB, Susanne Ditlevsen, Eva Garde, Rikke G. Hansen, Adeline L. Samson, Alexander S. Conrad, Mads Peter Heide-Jørgensen. Stuck in a corner: Anthropogenic noise threatens narwhals in their once pristine Arctic habitat. *Science Advances*. 2023; **9**: eade0440. doi: 10.1126/sciadv.ade0440

10. Stacey PJ, Hvengaard GT. Habitat use and behaviour of Irrawaddy dolphins (Orcaella brevirostris) in the Mekong River of Laos. *Aquatic Mammals*. 2002; **28**(1): 1-13.

11. Richard G, Filatova OA, Samarra FIP *et al.* Icelandic herring-eating killer whales feed at night. *Mar Biol*. 2017; **164**(2): 32. doi: 10.1007/s00227-016-3059-8

12. Newman K, Springer AM. Nocturnal activity by mammal‐eating killer whales at a predation hot spot in the Bering Sea. *Marine Mammal Science*. 2008; **24**(4): 990-999. doi: 10.1111/j.1748-7692.2008.00236.x

13. Baird RW, Borsani JF, Hanson MB *et al.* Diving and night-time behavior of long-finned pilot whales in the Ligurian Sea. *Marine Ecology Progress Series*. 2002; **237**: 301-305. doi: 10.3354/meps237301

14. Barile C, Berrow S, Parry G *et al.* Temporal acoustic occurrence of sperm whales Physeter macrocephalus and long-finned pilot whales Globicephala melas off western Ireland. *Marine Ecology Progress Series*. 2021; **661**: 203-227. doi: 10.3354/meps13594

15. Pulis EE, Wells RS, Schorr GS *et al.* Movements and Dive Patterns of Pygmy Killer Whales (Feresa attenuata) Released in the Gulf of Mexico Following Rehabilitation. *Aquatic Mammals*. 2018; **43**(5): 555-567. doi: 10.1578/am.44.5.2018.555

16. Baumann-Pickering S, Roch MA, Wiggins SM *et al.* Acoustic behavior of melon-headed whales varies on a diel cycle. *Behav Ecol Sociobiol*. 2015; **69**(9): 1553-1563. doi: 10.1007/s00265-015-1967-0

17. West KL, Walker WA, Baird RW *et al.* Stomach contents and diel diving behavior of melon‐headed whales (Peponocephala electra) in Hawaiian waters. *Marine Mammal Science*. 2018; **34**(4): 1082-1096. doi: 10.1111/mms.12507

18. Deconto LS, Monteiro-Filho ELA. Day and night sounds of the Guiana dolphin, Sotalia guianensis (Cetacea: Delphinidae) in southeastern Brazil. *Acta ethologica*. 2015; **19**(1): 61-68. doi: 10.1007/s10211-015-0223-y

19. Temple AJ, Tregenza N, Amir OA *et al.* Spatial and Temporal Variations in the Occurrence and Foraging Activity of Coastal Dolphins in Menai Bay, Zanzibar, Tanzania. *PLoS One*. 2016; **11**(3): e0148995. doi: 10.1371/journal.pone.0148995

20. An X, Duan P, Li W *et al.* Biosonar activity of the Indo-Pacific humpback dolphin (Sousa chinensis) near the tunnel section of the world’s longest cross-sea bridge—the Hong Kong-Zhuhai-Macao Bridge—is negatively correlated with underwater noise. *Frontiers in Marine Science*. 2023; **10**: 1171709. doi: 10.3389/fmars.2023.1171709

21. Fang L, Lin W, Guo L *et al.* Monitoring Indo‐Pacific humpback dolphin occurrences in a highly urbanized estuary for informing conservation and management. *Aquatic Conservation: Marine and Freshwater Ecosystems*. 2020; **31**(3): 685-695. doi: 10.1002/aqc.3475

22. Pine MK, Wang K, Wang D. Fine‐scale habitat use in Indo‐Pacific humpback dolphins, Sousa chinensis, may be more influenced by fish rather than vessels in the Pearl River Estuary, China. *Marine Mammal Science*. 2016; **33**(1): 291-312. doi: 10.1111/mms.12366

23. Shaff JF, Baird RW. Diel and lunar variation in diving behavior of rough‐toothed dolphins (Steno bredanensis) off Kauaʻi, Hawaiʻi. *Marine Mammal Science*. 2021; **37**(4): 1261-1276. doi: 10.1111/mms.12811

24. Soldevilla MS, Wiggins SM, Hildebrand JA. Spatio-temporal comparison of Pacific white-sided dolphin echolocation click types. *Aquatic Biology*. 2010; **9**: 49-62. doi: 10.3354/ab00224

25. Kanes KSJ, Dosso SE, Insua TL *et al.* Seasonal and diel patterns in Pacific white-sided dolphin (Lagenorhynchus obliquidens) pulsed calls near Barkley Canyon. *Marine Mammal Science*. 2023: 1-16. doi: 10.1111/mms.13055

26. Soldevilla MS, Wiggins SM, Hildebrand JA. Spatial and temporal patterns of Risso's dolphin echolocation in the Southern California Bight. *J Acoust Soc Am*. 2010; **127**(1): 124-132. doi: 10.1121/1.3257586

27. Giorli G, Au WW, Ou H *et al.* Acoustic detection of biosonar activity of deep diving odontocetes at Josephine Seamount High Seas Marine Protected Area. *J Acoust Soc Am*. 2015; **137**(5): 2495-2501. doi: 10.1121/1.4919291

28. Benoit-Bird KJ, Southall BL, Moline MA. Dynamic foraging by Risso’s dolphins revealed in four dimensions. *Marine Ecology Progress Series*. 2019; **632**: 221-234. doi: 10.3354/meps13157

29. Dede A, Öztürk AA, Akamatsu T *et al.* Long-term passive acoustic monitoring revealed seasonal and diel patterns of cetacean presence in the Istanbul Strait. *Journal of the Marine Biological Association of the United Kingdom*. 2013; **94**(6): 1195-1202. doi: 10.1017/s0025315413000568

30. Elliott RG, Dawson SM, Henderson S. Acoustic monitoring of habitat use by bottlenose dolphins in Doubtful Sound, New Zealand. *New Zealand Journal of Marine and Freshwater Research*. 2011; **45**(4): 637-649. doi: 10.1080/00288330.2011.570351

31. Gauger MFW, Romero-Vivas E, Peck MA *et al.* Seasonal and diel influences on bottlenose dolphin acoustic detection determined by whistles in a coastal lagoon in the southwestern Gulf of California. *PeerJ*. 2022; **10**: e13246. doi: 10.7717/peerj.13246

32. Scott MD, Chivers SJ. Movements and diving behavior of pelagic spotted dolphins. *Marine Mammal Science*. 2009; **25**(1): 137-160. doi: 10.1111/j.1748-7692.2008.00241.x

33. Baird RW, Ligon AD, Hooker SK *et al.* Subsurface and nighttime behaviour of pantropical spotted dolphins in Hawai'i. *Canadian Journal of Zoology*. 2001; **79**(6): 988-996. doi: 10.1139/z01-070

34. McElligott MM, Lammers MO. Investigating Spinner Dolphin (Stenella longirostris) Occurrence and Acoustic Activity in the Maui Nui Region. *Frontiers in Marine Science*. 2021; **8**: 703818. doi: 10.3389/fmars.2021.703818

35. Howe M, Lammers MO. Investigating the Diel Occurrence of Odontocetes around the Maui Nui Region Using Passive Acoustic Techniques1. *Pacific Science*. 2021; **75**(1): 147–161. doi: 10.2984/75.1.7

36. Perrin WF, Robertson KM, Walker WA. Diet of the striped dolphin, Stenella coeruleoalba, in the eastern tropical Pacific Ocean. *NOAA Technical Memorandum NMFS-SWFSC*. 2008; **418**: 1-26.

37. Goold JC. A Diel Pattern in Vocal Activity of Short‐Beaked Common Dolphins, Delphinus Delphis. *Marine Mammal Science*. 2006; **16**(1): 240-244. doi: 10.1111/j.1748-7692.2000.tb00915.x

38. Leeney R. Using Static Acoustic Monitoring to Describe Echolocation Behaviour of Heaviside’s Dolphins (Cephalorhynchus heavisidii) in Namibia. *Aquatic Mammals*. 2011; **37**(2): 151-160. doi: 10.1578/am.37.2.2011.151

39. Zein B, Woelfing B, Dahne M *et al.* Time and tide: Seasonal, diel and tidal rhythms in Wadden Sea Harbour porpoises (Phocoena phocoena). *PLoS One*. 2019; **14**(3): e0213348. doi: 10.1371/journal.pone.0213348

40. Nuuttila HK, Bertelli CM, Mendzil A *et al.* Seasonal and diel patterns in cetacean use and foraging at a potential marine renewable energy site. *Mar Pollut Bull*. 2018; **129**(2): 633-644. doi: 10.1016/j.marpolbul.2017.10.051

41. Osiecka AN, Jones O, Wahlberg M. The diel pattern in harbour porpoise clicking behaviour is not a response to prey activity. *Sci Rep*. 2020; **10**(1): 14876. doi: 10.1038/s41598-020-71957-0

42. Schaffeld T, Bräger S, Gallus A *et al.* Diel and seasonal patterns in acoustic presence and foraging behaviour of free-ranging harbour porpoises. *Marine Ecology Progress Series*. 2016; **547**: 257-272. doi: 10.3354/meps11627

43. Todd NRE, Jessopp M, Rogan E *et al.* Extracting foraging behavior from passive acoustic monitoring data to better understand harbor porpoise (Phocoena phocoena) foraging habitat use. *Marine Mammal Science*. 2022; **38**(4): 1623-1642. doi: 10.1111/mms.12951

44. Todd VLG, Pearse WD, Tregenza NC *et al.* Diel echolocation activity of harbour porpoises (Phocoena phocoena) around North Sea offshore gas installations. *Ices Journal of Marine Science*. 2009; **66**(4): 734-745. doi: 10.1093/icesjms/fsp035

45. Linnenschmidt M, Teilmann J, Akamatsu T *et al.* Biosonar, dive, and foraging activity of satellite tracked harbor porpoises (Phocoena phocoena). *Marine Mammal Science*. 2012; **29**(2): 77-97. doi: 10.1111/j.1748-7692.2012.00592.x

46. Brandt MJ, Hansen S, Diederichs A *et al.* Do man‐made structures and water depth affect the diel rhythms in click recordings of harbor porpoises (Phocoena phocoena)? *Marine Mammal Science*. 2014; **30**(3): 1109-1121. doi: 10.1111/mms.12112

47. Cardenas Hinojosa G, de la Cueva H, Gerrodette T *et al.* Distribution of the acoustic occurrence of dolphins during the summers 2011 to 2015 in the Upper Gulf of California, Mexico. *PeerJ*. 2020; **8**: e9121. doi: 10.7717/peerj.9121

48. Cheng Z, Li Y, Pine MK *et al.* Association between porpoise presence and fish choruses: implications for feeding strategies and ecosystem-based conservation of the East Asian finless porpoise. *Integr Zool*. 2023; **18**(1): 169-182. doi: 10.1111/1749-4877.12639

49. Ogawa M, Kimura SS. Variations in echolocation click characteristics of finless porpoise in response to day/night and absence/presence of vessel noise. *PLoS One*. 2023; **18**(8): e0288513. doi: 10.1371/journal.pone.0288513

50. Sasaki‐Yamamoto Y, Akamatsu T, Ura T *et al.* Diel changes in the movement patterns of Ganges River dolphins monitored using stationed stereo acoustic data loggers. *Marine Mammal Science*. 2012; **29**(4): 589-605. doi: 10.1111/j.1748-7692.2012.00590.x

51. Yamamoto Y, Akamatsu T, da Silva VMF *et al.* Local habitat use by botos (Amazon river dolphins, Inia geoffrensis) using passive acoustic methods. *Marine Mammal Science*. 2015; **32**(1): 220-240. doi: 10.1111/mms.12252

52. Paitach RL, Amundin M, Teixeira G *et al.* Echolocation variability of franciscana dolphins (Pontoporia blainvillei) between estuarine and open-sea habitats, with insights into foraging patterns. *J Acoust Soc Am*. 2021; **150**(5): 3987. doi: 10.1121/10.0007277
